# Effects of drospirenone and ethinylestradiol tablets (II) combined with metformin on the composition of gut microbiota in polycystic ovary syndrome with insulin resistance

**DOI:** 10.3389/fendo.2025.1581504

**Published:** 2025-10-13

**Authors:** Yanglu Liu, Qingmei Wang, Lingling Han, Xue Shao, Xin Wang, Xin Liu, Jiayi Wang, Shuang Luo, Baiyu Lu

**Affiliations:** ^1^ Department of Obstetrics and Gynecology, Suining Central Hospital, Suinig, Sichuan, China; ^2^ School of Medical and Life Sciences, Chengdu University of Traditional Chinese Medicine, Chengdu, Sichuan, China

**Keywords:** pcos, insulin resistance, gut microbiota, 16S rDNA, drospirenone and ethinylestradiol tablets (II), metformin

## Abstract

**Objectives:**

To explore the relationship between polycystic ovary syndrome with insulin resistance (PCOS-IR) and gut microbiota by analyzing the composition characteristics of gut microbiota in PCOS without or with Insulin resistance (IR) and the changes before and after treatment.

**Methods:**

Women with PCOS were recruited and divided into polycystic ovary syndrome without insulin resistance (PCOS-NIR) group and PCOS-IR group. The PCOS-NIR group was given oral drospirenone and ethinylestradiol tablets (II), and the PCOS-IR group was given oral drospirenone and ethinylestradiol tablets (II) combined with metformin hydrochloride tablets. The course of treatment was 3 months. Baseline data, blood and stool samples were collected before and after treatment. All those stool samples were used for 16S rDNA sequencing to analyze the composition of gut microbiota in each group and their correlation with biochemical parameters.

**Results:**

1. Baseline data and biochemical parameters: before treatment, body mass index (BMI) in PCOS-NIR group was less than that in PCOS-IR group(*P* < 0.05). After treatment, CHO levels decreased in PCOS-NIR group (*P* < 0.05); FINS, LDL-C and HOMA-IR decreased and HDL-C levels increased in PCOS-IR group (*P* < 0.05). 2. Characteristics of gut microbiota: before treatment, there was no significant difference in α and β diversity between PCOS-NIR group and PCOS-IR group. The gut microbiota both of the two groups were mainly composed of *Firmicutes*, *Fusobacteriia, Acidimicrobiia, Oscillospirales, Lachnospiracea, Ruminococcaceae*. The relative abundance of *Acidimicrobiia* in PCOS-IR group was higher than that in PCOS-NIR group (*P* < 0.05). The relative abundance of *Fusobacteriu* in the PCOS-IR group was lower than that before treatment (*P* < 0.05). 3. Correlation between gut microbiota and biochemical parameters: before treatment, *Acidimicrobiia* was positively correlated with TG level in the PCOS-NIR group (*P* < 0.05); *Acidimicrobiia* was positively correlated with HOMA-IR in the PCOS-IR group (*P* < 0.05). After treatment, *Fusobacterium* in PCOS-IR group was negatively correlated with HDL-C level (*P* < 0.05).

**Conclusion:**

1. There is a difference in the relative abundance of *Acidimicrobiia* in the gut between PCOS-NIR and PCOS-IR patients before treatment, which is positively correlated with HOMA-IR, suggesting that *Acidimicrobiia* may be involved in the occurrence of PCOS-IR. 2. Drospirenone and ethinylestradiol tablets (II) combined with metformin tablets can change the gut microbiota relative abundance of patients with PCOS-IR, which may be related to the improvement of host lipid and glucose metabolism.

## Introduction

Polycsytic Ovarian Syndrome (PCOS) is a common reproductive endocrine disease in gynecology, which often occurs in women of childbearing age. It is characterized by excessive androgen, persistent anovulation, and polycystic ovarian changes, often accompanied by Insulin Resistance (IR) and obesity, which affects the life quality, physical and mental health of patients (1, 2), and is one of the causes of female infertility (3, 4).PCOS patients have an increased risk of developing type 2 diabetes, coronary heart disease, atherosclerotic dyslipidemia, cerebrovascular disease (5), anxiety, depression (6), and have an increased morbidity of long-term complications such as endometrial hyperplasia and endometrial cancer (7, 8). Women with PCOS have an increased risk of gestational diabetes mellitus, hypertensive disorders complicating pregnancy, and other complications (9). The increasing morbidity of PCOS has raised concern among researchers. According to statistics, in recent years, the global incidence of PCOS is about 5%-15% (10), and the morbidity of PCOS in women of childbearing age in China is about 5.6% (11). The etiology of PCOS is complex, it is not clear, may be caused by genetic, environmental factors (12). A study suggested the pathogenesis of PCOS may be associated with IR and decreased insulin sensitivity (13). However, among the Chinese Han women with PCOS, the morbidity of IR is about 56.3%, and the prevalence of decreased insulin sensitivity is about 30.3% (14). Clinical studies have found that oral short-acting contraceptives combined with metformin can significantly improve glucose metabolism and regulate menstrual cycle in PCOS IR patients (15, 16). Oral contraceptives such as drospirenone and ethinylestradiol tablets (II) and ethinylestradiol and cyproterone tablets can relieve the syndrome of hyperandrogenism and regulate the menstrual cycle of PCOS patients, but drospirenone and ethinylestradiol tablets (II) is better than ethinylestradiol and cyproterone tablets in PCOS treatment (17). Metformin is a first-line drug for the treatment of type 2 diabetes and is also the most widely used insulin sensitizer in the treatment of PCOS, which is usually used to improve metabolic symptoms of PCOS patients and may also restore ovulation (18). Studies have found that oral drospirenone and ethinylestradiol tablets (II) combined with metformin can regulate the menstrual cycle of PCOS patients, relieve IR and improve abnormal lipid and glucose metabolism (19, 20). Therefore, it is necessary to use oral contraceptives combined with metformin in the treatment of PCOS patients with IR (21).The main treatment method of PCOS in the world is to improve reproductive endocrine and metabolic symptoms, such as hyperandrogenic symptoms, menstrual cycle regulation, infertility, etc (22). 2018 PCOS guidelines recommend that the patients can take oral contraceptives pills 3 to 6 month (23). During the treatment of patients with symptoms can improve, but some patients menstrual disorder relapse after drug withdrawal. The treatment of PCOS requires long-term management, and it is necessary to further explore the pathogenesis and new treatment methods of PCOS. The normal human gut is home to about 500 to 1000 species of bacteria, which encode a total of more than 100 times the number of human genes. These microbial genes can guide the synthesis of a variety of bioactive proteins and enzymes, and then participate in a wide range of host physiological and biochemical reactions. Therefore, the gut microbiome is known as the “second genome” of the human body (24). These bacteria are also distributed outside the digestive tract, such as respiratory tract, reproductive tract and other parts, which can serve as biochemical barriers and participate in the host nutrient catabolism, digestion and absorption, immune regulation and other processes, and play an important role in maintaining the host health.With the development of gene sequencing technology in gut microbiota research, scholars have found a certain relationship between some metabolic diseases (such as obesity, type 2 diabetes and cardiovascular disease) and gut microbiota (25, 26). Factors such as hyperandrogenism, IR, obesity, metabolic disorders and unhealthy diet associated with PCOS can damage intestinal flora and its metabolites, thereby worsening the pathological process of PCOS and forming a malignant cycle (27). Compared with healthy women, PCOS patients have different gut microbiota: *Akkermensia* and *Ruminococcaceae* are decreased, and *Bacteroides*, *Escherichia* coli and *Shigella* are increased (28). Qi X speculated that the increased relative abundance of *Bacteroides* in PCOS patients leads to intestinal metabolite disorder, which may have a potential pathological role in PCOS (29). The relationship between PCOS and gut microbiota is complex. Animal experiments and clinical studies have found that healthy mice colonized the feces of PCOS women appear to have a PCOS-like phenotype (30), while the colonization of healthy intestinal microorganisms, supplementation of probiotics or specific intestinal metabolites can prevent the occurrence of PCOS-like traits in mice (28). On the other hand, supplementation of probiotics can improve hyperandrogenism, inflammation and oxidative stress in PCOS patients (31). In these cases, the regulation of gut microbiota may become a new treatment option for PCOS.

PCOS pathologic physiology and clinical manifestations are heterogeneity. Since the 1980s, IR has been known as significant clinical symptom of PCOS (32), but some PCOS patients have no IR. Gut microbiota dysbiosis may exacerbate Insulin Resistance (IR): The double-edged sword effect of short-chain fatty acids (SCFAs): SCFAs (e.g., butyrate, propionic acid) produced by probiotic fermentation of dietary fiber could have improved insulin sensitivity and energy metabolism.However, the production of SCFAs may be unbalanced under the dysbacteriosis associated with PCOS. Metabolites produced by some harmful bacteria, such as endotoxin LPS, enter the blood circulation and trigger low-grade chronic inflammation, interfere with insulin signaling pathway, and directly promote the occurrence of IR. The results of current studies on PCOS and gut microbiota are different, and the causality between them has not been clarified. Further studies are needed to observe the composition of gut microbiota in PCOS patients and explore the role of gut microbiota in the occurrence and development of PCOS. In this study, 16S rDNA sequencing technology was used to analyze the composition of gut microbiota in patients with PCOS-NIR and PCOS-IR, and to observe the effect of oral drospirenone and ethinylestradiol tablets (II) or combined with metformin tablets on gut microbiota in these PCOS patients. The correlation between the structural changes of intestinal flora in phylum, class, order, family, genus, species and the characteristics of PCOS was also explored before and after treatment.

## Subjects and methods

A total of 40 women with PCOS who were first diagnosed in Suining Central Hospital from November 2021 to November 2023 were recruited in this study.

### Inclusion criteria

(1) female aged 18–45 years old; (2) The 2003 Rotterdam criteria for PCOS: i oligo-ovulation or anovulation; ii hyperandrogenic manifestations and/or hyperandrogenism; iii ovarian polycystic changes: ultrasound reports suggested that the number of follicles in unilateral or bilateral ovaries with a diameter of 2–9 mm was greater than 12 and/or the ovarian volume was greater than 10ml; At least two of the above criteria were met and other causes of hyperandrogenism, such as Cushing’s syndrome, congenital adrenal hyperplasia, androgen-secreting tumors, were excluded.

### Exclusive criteria

(1) pregnant and lactating women; (2) use of antibiotics, oral contraceptives, and hypoglycemic drugs within the past three months; (3) patients who had undergone gastrointestinal surgery or received fasting within the past three months; (4) smoking; (5) patients with primary diseases of the blood system, endocrine system, cardiovascular and cerebrovascular systems; (6) abnormal liver function, renal function, contraindications or allergic constitution.

IR was detected by the homeostasis model assessment method (HOMA-IR). HOMA-IR=FINS×FPG/22.5, HOMA-IR≥2.69 is IR (33). All these patient were divided into PCOS-NIR group (n=20) and PCOS-IR group (n=20). The patients in PCOS-NIR group were given drospirenone and ethinylestradiol tablets (II) for 3 months when the first day of menstrual cycle. The patients in PCOS-IR group were given drospirenone and ethinylestradiol tablets (II) combined with metformin hydrochloride tablets for 3 months when the first day of menstrual cycle. Biochemical parameters such as Fasting plasma glucose (FPG), Fasting insulin (FINS), Luteinizing hormone (LH), Follicle Stimulating Hormone (FSH), Triglycerides (TG), Low density lipoprotein cholesterol (LDL-C) and High density lipoprotein cholesterol (HDL-C) were detected before and after treatment. At the same time, patients stool samples were collected respectively, all those stool samples were used for 16S rDNA sequencing. Patients in both groups were followed up by telephone for 3 consecutive months after drug withdrawal, which were consulted by telephone about the menstruation and pregnancy.

### Stool samples collection

Stool samples were collected before treatment and after 3 months of treatment, with each collection collected between 3 and 7 days after menstruation was clean.Three days before stool collection, the patient had a light diet to avoid diarrhea and constipation. About 5 grams of stool samples were collected each time, then immersed in the stool sample storage tube, thoroughly mixed and quickly stored in the refrigerator at -80 °C.

### 16S rDNA sequencing

Cetyltrimethylammonium bromide method (CTAB) was used to extract genomic DNA from the collected samples. (2) The purity and concentration of DNA were detected by agarose gel electrophoresis. (3) Library construction: appropriate samples were taken in a centrifuge tube, diluted to 1ng/μl with sterile water, and the diluted genomic DNA was used as a template. According to the selection of sequencing region, specific primers with Barcode were used for PCR with high efficiency and high fidelity. Primers corresponding to the region: 16S V4 region primer 515F-806R; The primer of 16S V3-V4 region was 338F-806R. The primer of 18S V4 region was 528F-706R. The primer of 18S V9 region was 1380F-1510R. The primers of ITS1 region were ITS1F-ITS2. The primers of ITS2 region were: ITS2-3F– ITS2-4R; The PCR products were detected by electrophoresis on 2% agarose gel. library quality inspection; Raw data obtained by IIIumina MiSeq sequencing.

### Statistical methods

This study used SPSS 26.0 to analysis, with a mean ± standard deviation. Wilcoxon rank sum test was used for statistical analysis of gut microbiota data. The correlation between intestinal flora and biochemical indicators was analyzed by pearson correlation analysis using rcorr function of Hmisc package. *P* < 0.05 was considered statistically significan.

### Informed consent and ethical approval

The study strictly adhered to the principles of the Declaration of Helsinki, and the patients were explained in relevant aspects before enrollment and signed the informed consent form.

## Results

### Basic information and biochemical indicators

A total of 40 patients were enrolled in this study, and there were 19 patients in the two groups who did not follow the doctor’s advice (the course of treatment was less than 3 months). Finally, only 21 patients were left (11 in the PCOS-NIR group and 10 in the PCOS-IR group). Before treatment, there was no significant difference in age and height between the PCOS-NIR group and the PCOS-IR group (*P*>0.05); Although body weight, BMI, waist circumference, hip circumference and WHR in the PCOS-NIR group were lower than those in the PCOS-IR group, only BMI difference was statistically significant (27.12 ± 2.53 vs30.94 ± 4.69,P=0,01), as shown in **Table 1**. The levels of FINS, FPG, HOMA-IR and LDL-C in the PCOS-NIR group were lower than those in the PCOS-IR group(8.03 ± 1.87 vs 24.08 ± 10.95 *P* = 0.00; 5.40 ± 0.46 vs 6.01 ± 1.20 *P* = 0.04; 1.91 ± 0.43 vs 6.57 ± 3.78 P = 0.00 3.06 ± 1.03 vs 4.25 ± 1.26 *P* = 0.03). There were no significant differences in the levels of HDL-C, CHO, TG, FSH, LH, E2, T, Pro and PRL between the PCOS-NIR and PCOS-IR groups (P>0.05), as shown in **Table 2** After treatment, there were no significant differences in body weight, BMI, waist circumference, hip circumference and WHR of the two groups than those of before treatment (*P*>0.05), as shown in **Table 1**. The CHO level in the PCOS-NIR group was lower than that before treatment (4.25 ± 1.62 vs 3.93 ± 1.05, P = 0.01). The levels of FINS, HOMA-IR and LDL-C in the PCOS-IR group were lower than those before treatment (24.08 ± 10.95 vs 18.14 ± 5.10, P = 0.028; 6.57 ± 3.78 vs 4.42 ± 1.13, P = 0.04; 4.25 ± 1.26 vs 3.01 ± 1.23, P = 0.01). The HDL-C level was higher than that before treatment (1.35 ± 0.66 vs 1.56 ± 0.42, P = 0.02), as shown in **Table 2**.

**Table 1:** General parameters of subjects [ $\bar{x}$ ± *s*].

Parameters	PCOS-NIR (n=20)	PCOS-NIR treated (n=11)	PCOS-IR (n=20)	PCOS-IR treated (n=10)
age(year)	26.30 ± 5.03	26.64 ± 5.61	26.10 ± 4.98	27.00 ± 4.19
height(cm)	158.55 ± 5.91	157.55 ± 6.82	158.35 ± 5.30	158.40 ± 5.34
weight(kg)	68.30 ± 7.16	70.09 ± 5.52	77.85 ± 11.39	75.90 ± 8.97
BMI	27.12 ± 2.53^a^	28.47 ± 2.51	30.94 ± 4.69^a^	29.50 ± 4.29
waistline(cm)	82.15 ± 9.35	85.55 ± 8.36	95.60 ± 8.52	93.00 ± 1.79
hipline(cm)	97.70 ± 6.10	99.73 ± 5.24	105.70 ± 7.75	103.30 ± 3.56
WHR	0.83 ± 0.05	0.85 ± 0.05	0.90 ± 0.04	0.89 ± 0.43

PCOS-NIR group compared with PCOS-IR group, ^a^P< 0.05.

**Table 2:** Biochemical parameters [ $\bar{x}$ ± *s*].

Parameters	PCOS-NIR (n=20)	PCOS-NIR treated (n=11)	PCOS-IR (n=20)	PCOS-IR treated (n=10)
FINS(mIU/L)	8.03 ± 1.87^a^	8.05 ± 1.82	24.08 ± 10.95^ac^	18.14 ± 5.10^c^
FPG(mmol/L)	5.40 ± 0.46^a^	5.38 ± 0.46	6.01 ± 1.20^a^	5.52 ± 0.41
HOMA-IR	1.91 ± 0.43^a^	1.92 ± 0.42	6.57 ± 3.78^ac^	4.42 ± 1.13^c^
LDL-C(mmol/L)	3.06 ± 1.03^a^	2.88 ± 0.68	4.25 ± 1.26^ac^	3.01 ± 1.23^c^
HDL-C(mmol/L)	1.49 ± 0.32	1.66 ± 0.25	1.35 ± 0.66^c^	1.56 ± 0.42^c^
CHO(mmol/L)	4.25 ± 1.62^b^	3.93 ± 1.05^b^	4.70 ± 1.67	4.08 ± 1.31
TG(mmol/L)	1.97 ± 1.23	1.89 ± 1.21	1.92 ± 0.98	1.63 ± 0.61
LH (mIU/ml)	11.49 ± 8.86	11.52 ± 12.04	8.81 ± 3.97	8.48 ± 3.28
FSH (mIU/ml)	5.46 ± 1.65	4.46 ± 1.81	4.72 ± 1.59	6.03 ± 1.69
E^2^ (pg/ml)	55.26 ± 40.83	91.55 ± 57.59	67.44 ± 50.16	103.18 ± 65.16
T (nmol/L)	1.68 ± 0.50	1.43 ± 0.64	1.70 ± 0.40	1.63 ± 0.46
Pro (ng/ml)	1.11 ± 1.98	2.31 ± 3.29	1.36 ± 2.73	1.37 ± 1.95
PRL(ng/ml)	15.5 ± 8.83	20.90 ± 9.68	15.55 ± 8.83	15.69 ± 3.35

PCOS-NIR group compared with PCOS-IR group, ^a^ *P* < 0.05; Compared before and after treatment in the PCOS-NIR group, ^b^ *P* < 0.05; Compared before and after treatment in the PCOS-IR group, ^c^ *P* < 0.05;.

### Sequencing and analysis of gut microbiota

#### Gut microbiota sequencing results

**Figure 1:**
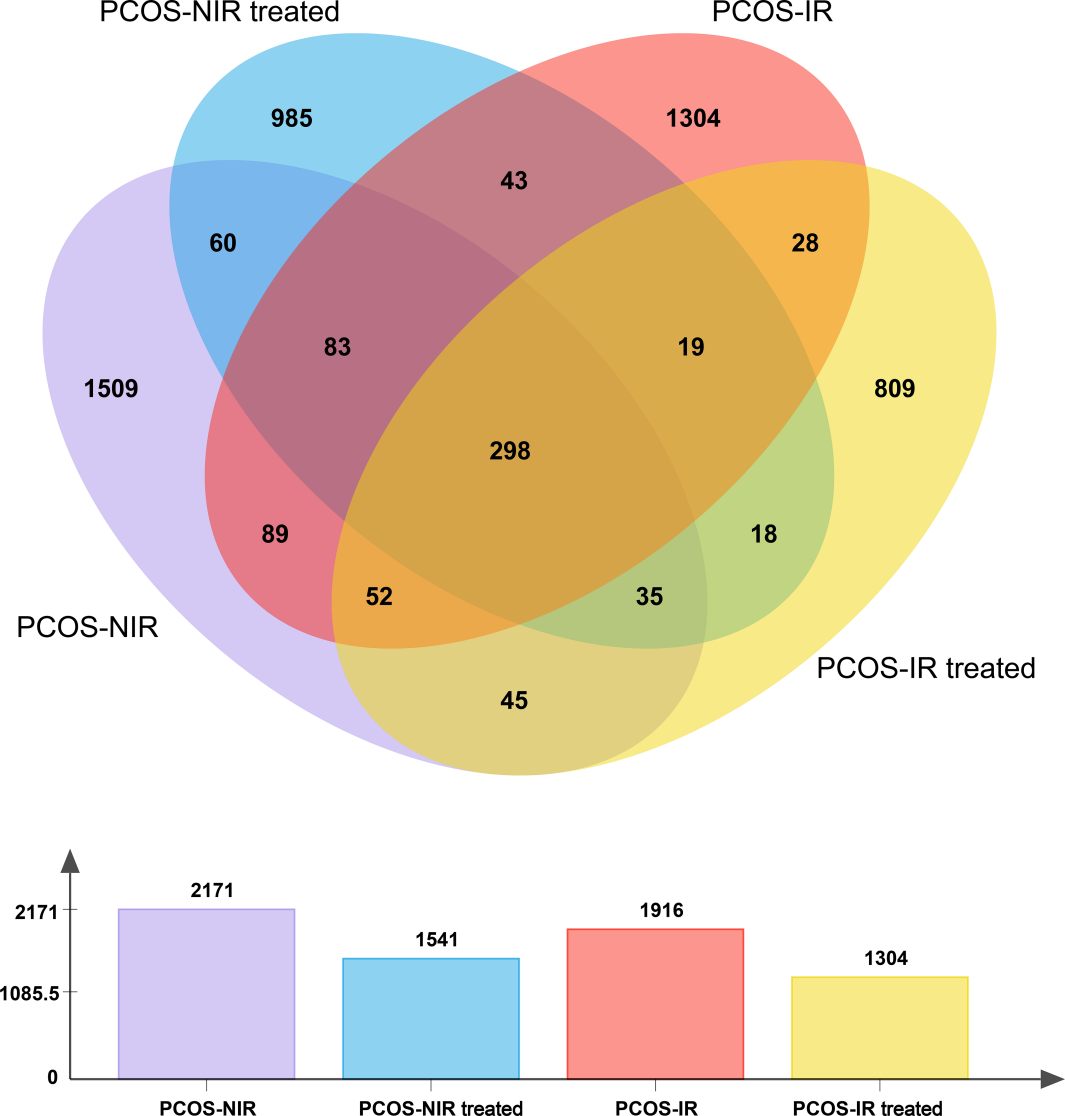
The number of ASVs.

According to the characteristics of the amplified 16S region, a small fragment library was constructed by double-end sequencing based on Illumina MiSeq sequencing platform. By splicing, filtering and denoising short sequences at both ends to generate Amplicon Sequence Varients (ASVs) representing sequence and abundance information. The species classification analysis and annotation were performed to obtain the basic abundance of the taxonomic lineage of each sample. The abundance information of ASVs could be used for the calculation of diversity index, the test of differences, and the statistical analysis of community structure at each taxonomic level. As shown in **Figure 1**, there were a total of 6932 ASVs in the stool samples of all patients. Before treatment, there were 2171 ASVs in the PCOS-NIR group and 1916 ASVs in the PCOS-IR group. After treatment, there were 1541 ASVs in the PCOS-NIR group and 1304 ASVs in the PCOS-IR group.

#### Analysis of gut microbiota sequence

**Figure 2:**
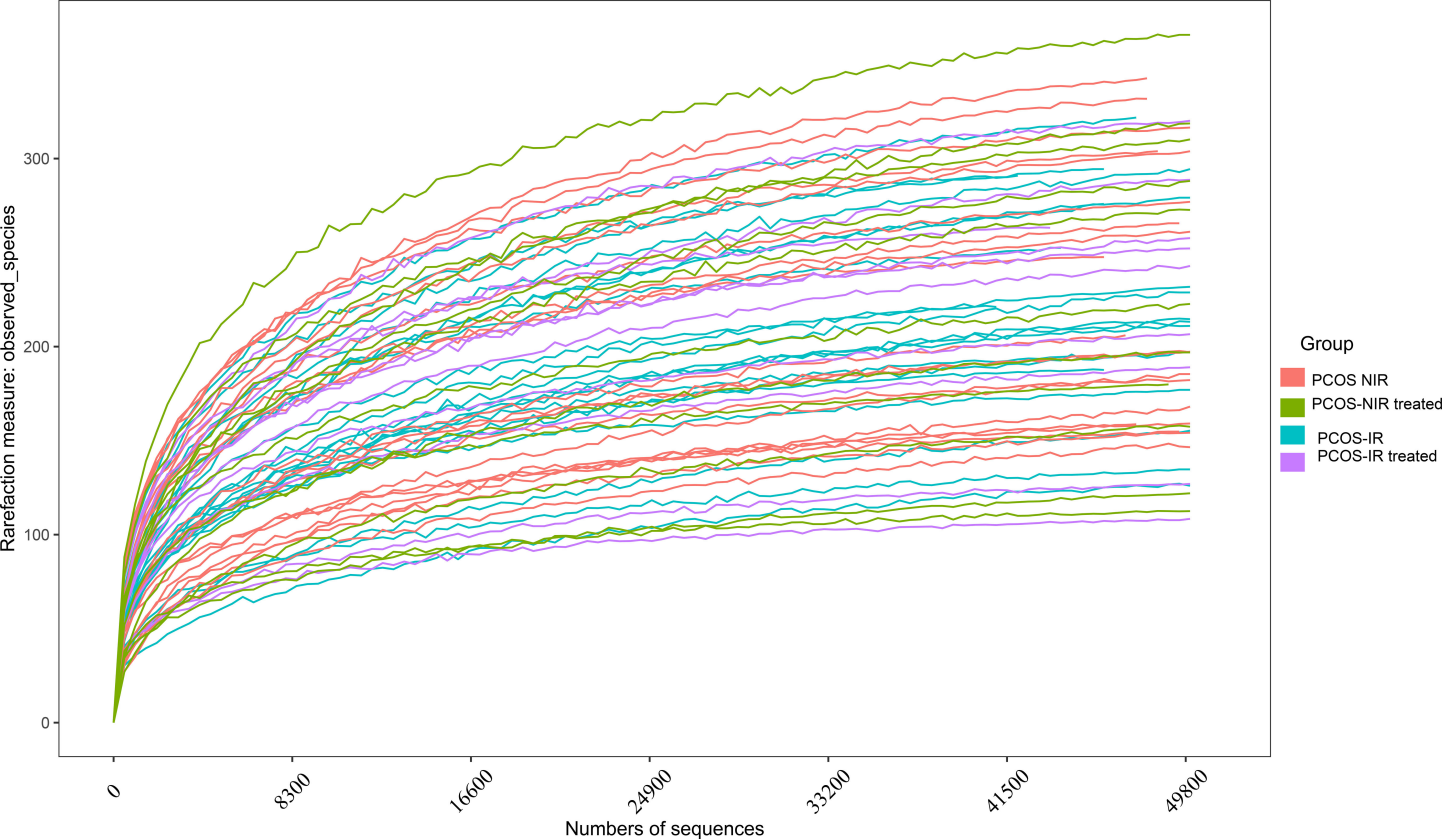
Rarefaction curve.

Rarefaction curve is used to indicate the sequencing of the sample data is required for subsequent analysis and indirectly reflect the species richness in the sample. In this study, the rarefaction curves of stool samples in each group gradually flattened, and the number of core species stopped increasing with the increase of sequence number. The sequencing data of this study were sufficient to meet the requirements of subsequent analysis, as shown in **Figure 2**.

#### Gut microbiota α diversity nanlysis

**Figure 3:**
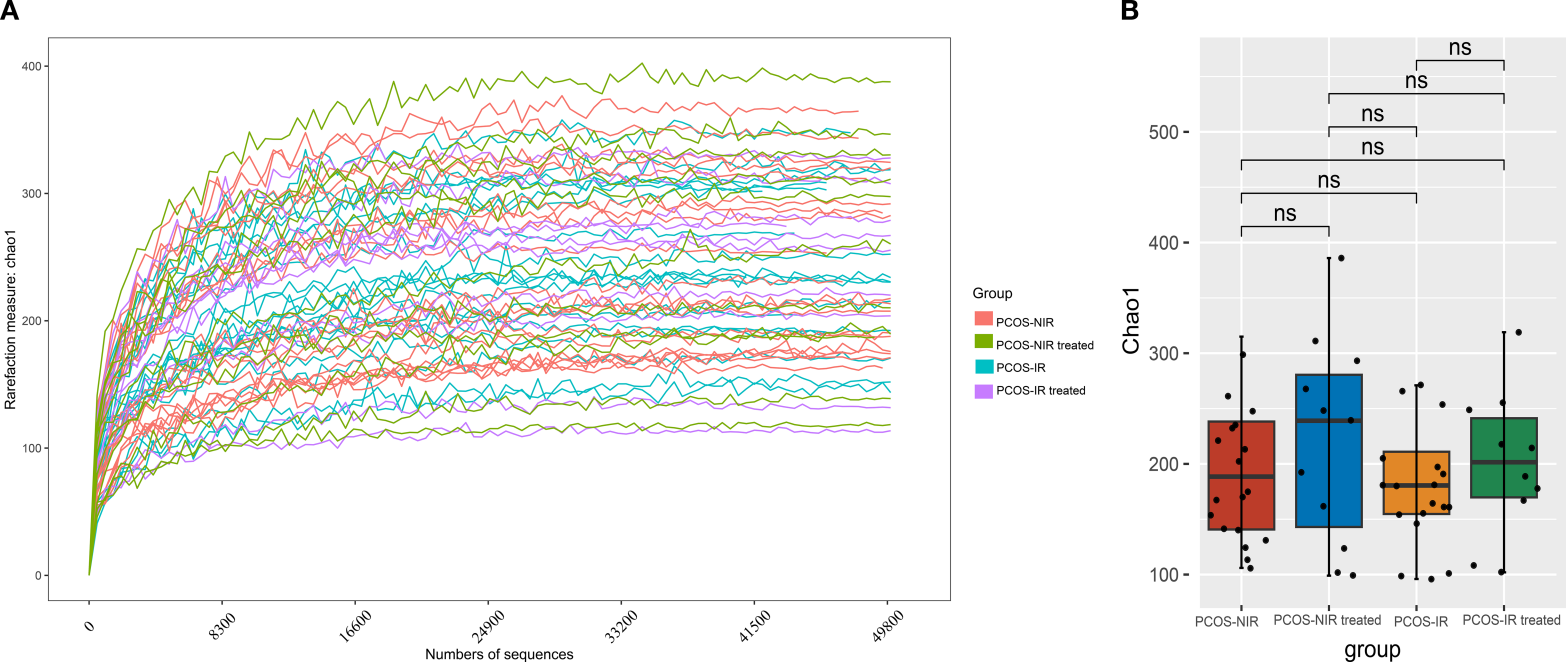
**(A)** Chao1 index curve. **(B)** Chao1 index analysis of gut microbiota.

Alpha diversity reflects the diversity of species within a single body. Chao1 index value can be combined with Wilcoxon rank sum test to analyze the difference of α diversity among samples of each group (34). As shown in **Figures 3A, B**, the ASVs were further analyzed gut microbiota α diversity through Chao 1 index and wilcoxon rank sum test. Before treatment, the α diversity of gut microbiota in the PCOS-NIR group was slightly higher than that in the PCOS-IR group, but the difference was not statistically significant. After treatment, the α diversity of gut microbiota in the PCOS-NIR group was higher than that before treatment, while the α diversity of gut microbiota in the PCOS-IR group was also higher than that before treatment, but both of these differences were not statistically significant.

#### Gut microbiota β diversity nanlysis

**Figure 4:**
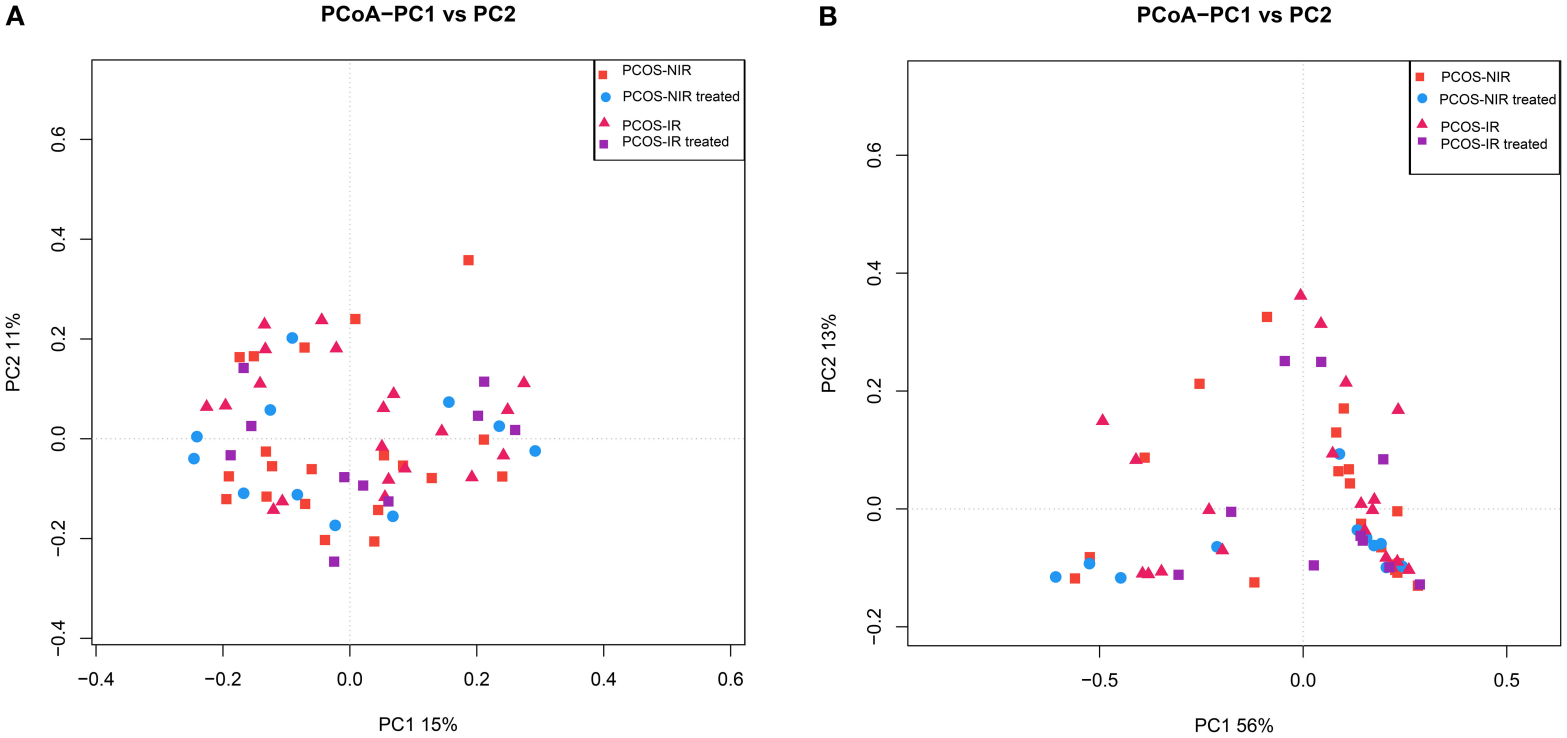
**(A)** PCoA analysis based on unweighted unifrac distance. **(B)** PCoA analysis based on weighted unifrac distance.

β diversity reflects the degree of species difference among individuals. The Unweighted and Weighted Unifrac distance matrices calculated based on QIIME2 can be analyzed by multivariate statistical method PCoA (35) to visually display the evolutionary similarity and difference of species in samples between groups. The closer the distance between the samples in each group, the more similar the species composition of the samples was. As shown in **Figures 4A, B**, there was no significant aggregation of gut microbiota species in the PCOS-NIR and PCOS-IR groups before treatment, and the composition of the microbiota was similar in both groups. After treatment, there was no significant change in the composition of fecal flora in the PCOS-NIR group and PCOS-IR group respectively.

### Gut microbiota relative abundance analysis

#### The relative abundance of gut microbiota in the phylum, class, order, family, genus, species

**Figure 5:**
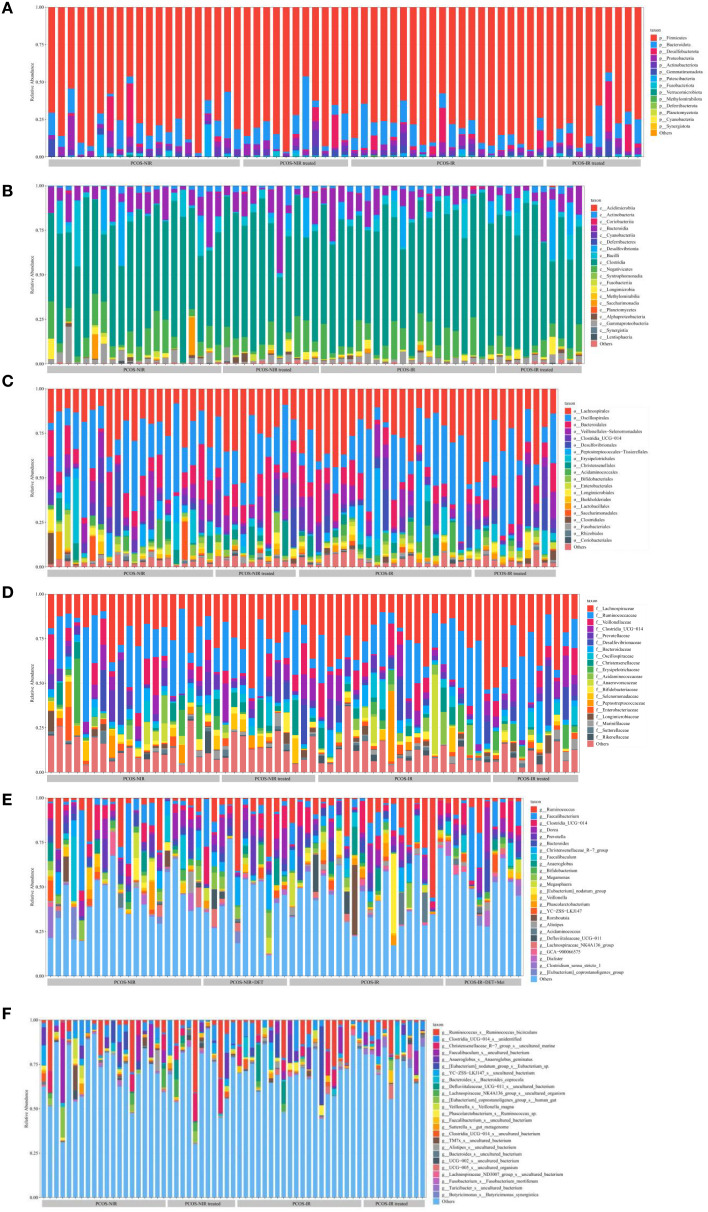
The relative abundance of gut microbiota on the phylum **(A)**, class **(B)**, order **(C)**, family**(D)**, genus **(E)** and species **(F)**.

According to the sequencing results, ASVs was used for species classification and annotation to obtain the relative abundance of the taxonomic lineage of each sample. Before treatment, the species richness of gut microbiota were similar between PCOS-NIR group and PCOS-IR group at each taxonomic level. At the phylum level, the dominant bacteria were *Firmicutes* (**Figure 5A**). At the class level, the dominant bacteria were *Fusobacteriia*, *Acidimicrobiia* (**Figure 5B**). At the order level, the dominant bacteria was: *Oscillospirales* (**Figure 5C**). At the family level, the dominant bacteria were: *Lachnospirillaceae*, *Ruminococcaceae* (**Figure 5D**). There was no obvious dominant flora at the genus and species levels, as shown in **Figures 5E, F**. After treatment, the species richness of gut microbiota in the PCOS-NIR group and PCOS-IR group had no significant change compared with that before treatment.

#### The difference in the relative abundance of gut microbiota

**Figure 6:**
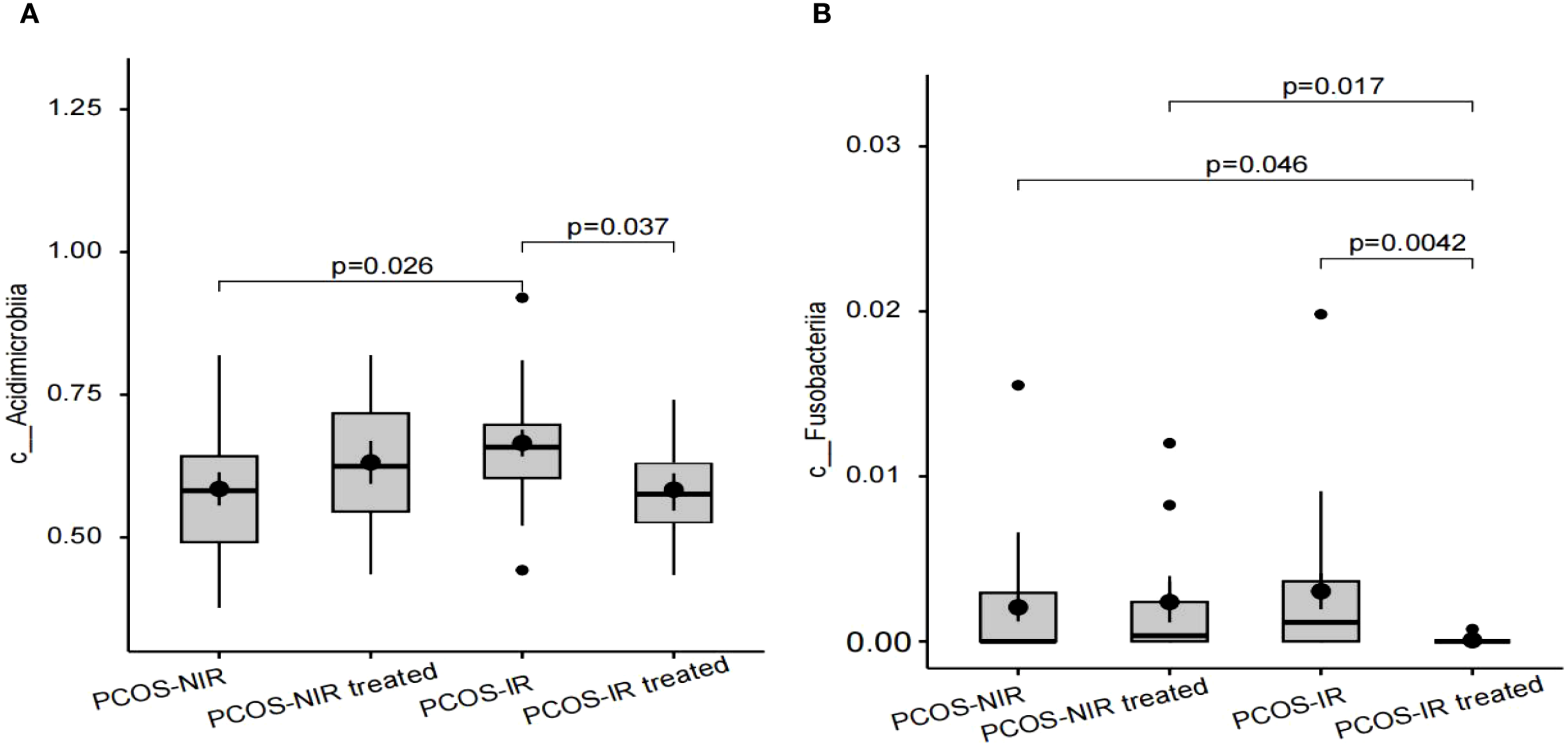
**(A)**Relative abundance of Acidimicrobiia. **(B)** Relative abundance of Fusobacteria.

The differences in the relative abundance of intestinal flora between the PCOS-NIR group and the PCOS-IR group before and after treatment were analyzed at the levels of phylum, class, order, family, genus and species, respectively, which found significant differences in the relative abundance of *Acidimicrobiia* and *Fusobacteriia* (*P* < 0.05), and there was no significant difference in the relative abundance of other taxa. Before treatment, the relative abundance of *Acidimicrobiia* in the PCOS-NIR group was lower than that in the PCOS-IR group (*P* < 0.05) (**Figure 6A**). After treatment, the relative abundance of *Acidimicrobiia* in the PCOS-NIR group was higher than that before treatment, but the difference was not statistically significant (*P>*0.05). In PCOS-IR group, the relative abundance of *Acidimicrobiia* was decreased, (*P* < 0.05) (**Figure 6A**), however, the relative abundance of *Fusobacteriia* was significantly lower than that before treatment (*P* < 0.05) (**Figure 6B**).

#### The correlation between dominant gut microbiota and clinical parameters

Before and after treatment, the correlation between the dominant gut microbiota of the PCOS-NIR group and PCOS-IR group and lipoglucose parameters was analyzed in the phylum, class, order, family and other levels.

**Figure 7:**
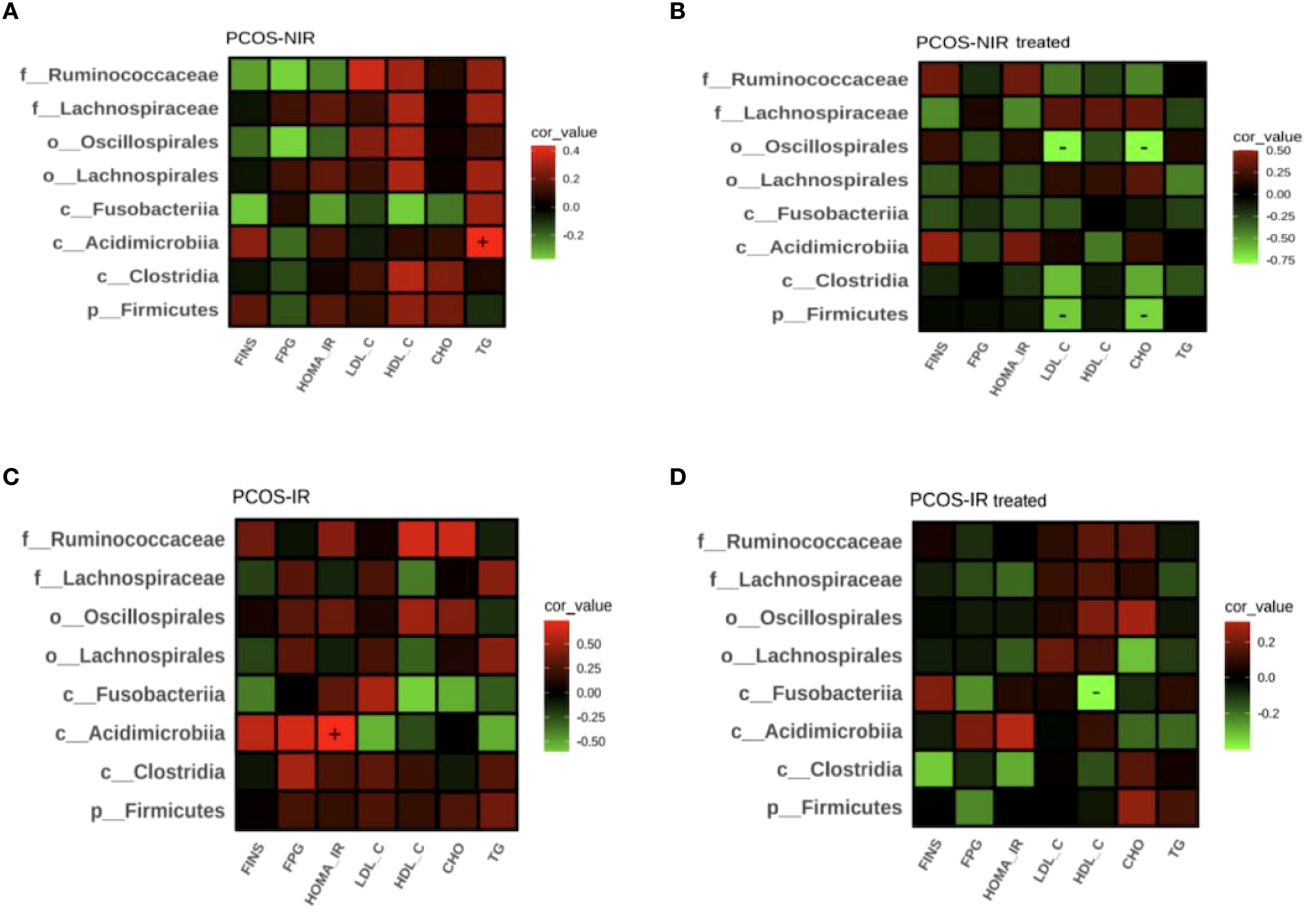
Correlation analysis chart of intestinal difference bacteria and clinical parameters of PCOS-NIR group **(A)**, PCOS-NIR treated group **(B)**, PCOS-IR group **(C)** and PCOS-IR treated group **(D)**.

Before treatment, the relative abundance of *Microacidobacteria* was positively correlated with TG level in the PCOS-NIR group (*P* < 0.05), in **Figure 7A**; In the PCOS-IR group, the relative abundance of *Microacidobacteria* was positively correlated with HOMA-IR (*P* < 0.05), in **Figure 7C**.

After treatment, the relative abundance of *Firmicutes* and *Frispirillum* in the PCOS-NIR group was negatively correlated with LDL-C and CHO levels, respectively (*P* < 0.05), in **Figure 7B**; In the PCOS-IR group, the relative abundance of *Fusobacteria* was negatively correlated with HDL-C level (*P* < 0.05), as shown in **Figure 7D**.

### Follow-up after drug withdrawal

Menstruation and pregnancy were followed up for 3 months after drug withdrawal (**Table 3**). In the PCOS-NIR group, 6 patients had menstruation in the first month after drug withdrawal, and 5 patients had menstrual disorder again. 4 patients had menstruation in the second month after drug withdrawal, and 7 patients had menstrual disorder again. In the third month after drug withdrawal, no menstruation occurred, 10 patients had menstrual disorder, and 1 patient became pregnant. In the PCOS-IR group, 5 patients had menstruation in the first month after drug withdrawal, and 5 patients had menstrual disorder again. In the second month after drug withdrawal, 3 patients had menstruation, 7 patients had menstrual disorder; In the third month after drug withdrawal, 2 patients had menstruation, 8 patients had menstrual disorder, and no pregnancy. In the two groups of patients with menstruation, the average length of menstrual period was 3–7 days. Some patients complained that the menstrual blood volume was less than that before treatment, but within the normal range.

**Table 3:** Follow-up of PCOS patients after drug withdrawal.

PCOS patient	PCOS-NIR(n=11)			PCOS-IR(n=10)		
Time after drug withdrawal (moth)	First	Second	Third	First	Second	Third
Menstruation(case)	6	4	0	5	3	2
pregnancy(case)	0	0	1	0	0	0

## Discussion

The etiology of PCOS is not clear, and may be related to lifestyle changes, genetic susceptibility, environmental changes and other factors. The pathological mechanism involves multiple systems, resulting in female reproductive endocrine dysfunction. IR is usually one of the metabolic disorder features of PCOS and is considered to be the main cause of PCOS (36). As a functional part of the human body, gut microbiota is known as the “new system” of the human body, and its homeostasis imbalance affects the health of the host (37). Clinical or animal experimental studies have found that intestinal flora imbalance is related to metabolic diseases (25, 26). However, the results of existing studies on PCOS and intestinal flora are not completely consistent, and the effect of short-acting contraceptives, insulin sensitizing agents and other drugs on the composition of intestinal flora in patients with PCOS is not clear. Therefore, this study used 16S rDNA sequencing to analyze the structural characteristics of gut microbiota in patients with PCOS-NIR and PCOS-IR, and the changes of gut microbiota after taking drospirenone and ethinylestradiol tablets (II) or combined with metformin tablets, so as to provide a theoretical basis for the regulation of intestinal flora as a new treatment for PCOS.

The morbidity of abnormal lipid metabolism in PCOS patients is about 70%, which is characterized by decreased HDL-C level and increased total cholesterol (38). However, Legro RS suggested that elevated LDL-C levels are characteristic of abnormal lipid metabolism in PCOS patients (39). The BMI and lipid levels of patients in the PCOS-IR group are higher than those in the PCOS-NIR group, which may be caused by IR increasing blood glucose level, converting excess glucose into fat, increasing blood lipid level and reducing fatty acid utilization. After treatment, CHO levels decreased in the PCOS-NIR group, drospirenone and ethinylestradiol tablets (II) can regulate cholesterol metabolism in the treatment of PCOS (40). The levels of FINS, HOMA-IR and LDL-C in PCOS-IR group are decreased, while the levels of HDL-C are increased. Drospirenone and ethinylestradiol tablets (II) and metformin tablets can improve the lipid and glucose metabolism of PCOS and reduce insulin resistance. Clinical studies have confirmed that the combination of the two drugs is effective in the treatment of PCOS with IR (41).

In recent years, it has been found that there are differences in the α diversity of gut microbiota between PCOS patients and healthy people (42). Torres PJ and Garcia-Beltran C et al. found that the α diversity of gut microbiota in PCOS patients decreased (43, 44). However, Mammadova G et al. found that the α diversity of PCOS patients increased (45). However, Fan Guanghui et al. studied the structural characteristics of gut microbiota in 10 patients with PCOS-IR and 11 patients with PCOS-NIR, and found that the diversity of intestinal flora in patients with PCOS-IR was lower than that in PCOS-NIR, and the β diversity was different (46). In this study, before treatment, there was no significant difference in the α and β diversity of gut microbiota between the PCOS-NIR group and the PCOS-IR group. After treatment, the α diversity of intestinal flora in the two groups showed an increasing trend, but there was no statistical significance. There also was no significant change in β-diversity. This result is similar to the research of Wang Xiaolian et al., who observed the changes of gut microbiota in 9 patients with PCOS-IR after Yusmin treatment (47). Deschasaux M believes that the diversity of gut microbiota may not be related to metabolic health, but can be explained by ethnic genetics, lifestyle or diet, etc., and individualized analysis may be a potentially important factor in the application of microbiome in disease exploration in the future (48).

In this study, the fecal samples of PCOS were enriched in *Firmicutes, Fusobacteriia*, *Acidimicrobiia*, *Oscillospirales*, *Lachnospiraceae* and *Ruminococcaceae*, while the enrichment was not obvious at the genus level and species level. Human gut microbiota mainly includes four categories: *Firmicutes*, *Bacteroidetes*, *Proteobacteria* and *Actinobacteria*. *Oscillospirales*, *Lachnospirillaceae* and *Ruminococcaceae* belong to *Firmicutes*, while *Fusobacteriia* belongs to *Bacteroidetes*. At other levels, due to the narrowing of the taxonomic relationship, the number of subtype species increased, so the dominant species was not obvious at the genus and species levels. He F et al. found that *Bacteroidetes* and *Firmicutes* were mostly enriched in the intestinal tract of insulin resistant PCOS (49). The enrichment of *Firmicutes*, intestinal short-chain fatty acid-producing *Lachnospiraceae* and *Ruminococcaceae* was also observed in the letrozole induced PCOS rat model (50, 51). In this study, the most obvious enrichment was *Firmicutes*, which accounted for the highest proportion in human gut microbiota (28). Some of them belong to beneficial bacteria and participate in carbohydrate metabolism. However, compared with healthy people, its relative abundance is reduced in PCOS patients (52). PCOS itself can aggravate inflammation and IR. When the abundance of *Firmicutes* is reduced, then production of short chain fatty acids (SCFAs) is also reduced, which weakens intestinal barrier function, increases pathogen invasion, promotes inflammation, and interferes with glucose metabolism, leading to IR (53). Regulating the proportion of *Firmicutes* in gut microbiota may improve lipid and glucose metabolism in PCOS.

The difference in the abundance of gut microbiota between PCOS patients and healthy patients is not consistent. Early clinical studies have found that PCOS patients have an increased abundance of *Bacteroides* and a decreased abundance of *Ruminococcaceae* (54). Later, Wang Xiaolian et al. also found that the abundance of *Bacteroides*, *Bifidobacterium*, *Fusobacteriia*, *Ruminococcaceae* and other bacteria decreased in PCOS patients with IR (48). However, Zhang J et al. found in the gut microbiota of PCOS patients that the abundance of *Faecalibacterium prausnitzii*, *Bifidobacterium*, *Parabacteroides* and *Fusobacteriia* increased in PCOS patients (53). The inconsistent results between PCOS and gut microbiota may be related to the heterogeneity of PCOS. IR is one of the significant endocrine characteristics of PCOS, but not all PCOS patients have IR. Before treatment, the relative abundance of gut microbiota of PCOS-NIR group was lower than that of PCOS-IR group, indicating that there was a difference in the composition ratio of gut microbiota between PCOS-NIR group and PCOS-IR group. Correlation analysis showed that *Acidimicrobiia* was positively correlated with TG level in the PCOS-NIR group, and *Acidimicrobiia* was also positively correlated with HOMA-IR in the PCOS-IR group. *Acidimicrobiia* are widely found in acidic environments. At present, there is a lack of research data on the correlation of PCOS. However, studies on patients with Alzheimer’s disease have found that *Acidimicrobiia* and *Christensenellaceae R-7 group* are related to adiponectin (55). Adiponectin is a peptide or protein secreted by fat cells, it can regulate insulin sensitivity and play an important role in regulating glucose metabolism. The secretion function of adipocytes in PCOS women is abnormal, and the level of adiponectin is decreased, which is correlated with metabolic indicators such as glucose, insulin and triglyceride (56). *Acidimicrobiia* may be involved in the synthesis and metabolism of adiponectin, and further regulate lipid and glucose metabolism and reduce insulin resistance in PCOS patients. After treatment, there was no significant difference in the relative abundance of intestinal flora in the PCOS-NIR group. However, it was found that the relative abundance of *Fusobacteriia* decreased in the PCOS-IR group after oral administration of drospirenone and ethinylestradiol tablets (II) combined with metformin tablets, and the relative abundance of *Fusobacteriia* was negatively correlated with HDL-C level. *Fusobacteriia* is a gram-negative spore free anaerobic bacteria, belonging to the opportunistic pathogenic bacteria, which distributes in the oral cavity, digestive tract and urogenital tract. Changing the residence site or the bacteria multiply too fast, and imbalance of the bacteria can cause the host disease. This bacteria has been found in oral diseases, coronary heart disease, gastrointestinal diseases, PCOS and other diseases (57, 58). The relative abundance of *Fusobacteriia* in the intestinal flora of PCOS patients increases, leading to the decrease of intestinal barrier function. Pathogens and pro-inflammatory substances such as lipopolysaccharide (LPS) reach various systems through the blood circulation, leading to pro-inflammatory changes in the body (59). Dyslipidemia and IR can also cause inflammation in PCOS patients through different mechanisms (60). The PCOS-IR patients in this study showed decreased HOMA-IR, increased HDL-C level, and decreased relative abundance of pro-inflammatory *Fusobacteriia* after treatment. The composition ratio of *Fusobacteriia* and *Acidimicrobiia* in gut microbiota may be used as a monitoring index to evaluate the efficacy of PCOS-IR treatment.

Clinical studies have confirmed the efficacy of drospirenone and ethinylestradiol tablets (II) and metformin in the treatment of PCOS patients (15–20). The study found that poor compliance in the treatment of patients with PCOS, especially those symptoms only characterized by irregular menstruation and no baby needs. This is also the main reason for dropout in this study. After treatment, the experimental sample size was 11 cases in the PCOS-NIR group and 10 cases in the PCOS-IR group. However, the rarefaction curve tended to be horizontal, and the sequencing data of core species were sufficient to meet the requirements of subsequent analysis. During the follow-up after drug withdrawal, it was found that most patients had regular menstruation during the treatment, but menstrual disorders recurred within a short period of drug withdrawal. The recurrence of menstrual disorders may be related to the treatment course. The degree of reproductive endocrine disorder in PCOS patients is different, and the treatment plan should be considered individually. An important limitation of this study was its relatively small final sample size (n=21) and high loss to follow-up rate (47.5%, 19/40). This inevitably introduces some potential bias and may affect the interpretation and generalization of the findings, which needs to be discussed in depth here.Reasons for loss to follow-up: most of the patients lost to follow-up had mild clinical symptoms; The long follow-up time led to loss of interest, and the cumbersome process of stool sample collection led to decreased compliance and unable to continue to participate. Unfortunately, we were not able to systematically collect detailed baseline characteristics or reasons for withdrawal for all lost participants. The small sample size significantly reduced the Statistical Power of the study. This meant that the study had limited power to detect real but small to moderate effect size differences in microbiota. Small sample sizes make the results more sensitive to Outliers or a few special individuals, and the study results are biased toward specific subtypes.The observed differences in microbiota abundance or association relationships may not be as stable as those in large sample studies, and their reproducibility needs to be verified in larger studies. PCOS itself is a highly heterogeneous syndrome. The small sample size and high rate of loss to follow-up that were observed in this study make it more difficult to capture a complete reflection of this heterogeneity at the gut microbiota level. The final analysis population may not have been sufficient to cover all key PCOS phenotypes, and thus the microbiota signatures found may be specific to specific subtypes rather than generalizable to all PCOS patients. In this study, those who remained had more severe clinical symptoms and better adherence, making the observed microbiota profile likely biased toward PCOS patients with insulin resistance. In conclusion, the small sample size and high rate of loss to follow-up are the core limitations of this study and may have introduced a selectivity bias, reduced statistical power and stability of the results, and limited the exploration of heterogeneity in PCOS. These factors call for caution in the interpretation of the current results and recognition of their preliminary and exploratory nature. Nonetheless, this study provides valuable data points and hypotheses for understanding the association between PCOS and gut microbiota, and the results can serve as a cornerstone for future larger, more well-designed studies, and highlight the importance of ensuring adequate sample size and minimizing loss to follow-up when conducting microbiome studies in a complex disease such as PCOS.

In conclusion, changes in gut microbiota may be involved in the occurrence of this disease. Drospirenone and ethinylestradiol tablets (II) combined with metformin tablets may enhance glucose metabolism, control inflammatory response, and improve lipid and glucose metabolism by regulating gut microbiota (61, 62). Other ways of regulating intestinal flora, such as synbiotics, can play an anti-obesity and improve insulin resistance by regulating human intestinal flora (63). Fecal microbiota transplantation not only has a significant effect on the intestinal microbiome, but also can improve insulin sensitivity by interfering with the physiological metabolism of host (64). Therefore, the regulation of gut microbita may become a new method for PCOS treatment, and the detection of gut biomarker-microbita may become an indicator for evaluating the efficacy of PCOS.

Although some association between gut microbiota and PCOS IR was observed in this study, there are limitations. Firstly, this study lacked a healthy control group. Thus, we were unable to make a direct comparison between the observed gut microbiome features and the microbiota structure of healthy individuals. Future studies involving age - and BMI-matched healthy controls will be able to more clearly elucidate the specific role of gut microbiota in the pathophysiology of PCOS. Secondly, the confounding effect of drugs must be fully considered. Some participants used medications such as metformin or oral contraceptives, which may modulate the composition and function of the gut microbiome. Despite attempts to adjust for the analyses, residual confounding may affect the interpretation of the results. At present, the research on the drug-microbiome-host interaction is in the rise, and its specific mechanism and long-term effects on host physiology are still being explored. Thus, it is possible that some of the microbiologic features observed in this study reflect, in part, the effects of drug therapy rather than disease states alone. Future prospective studies, especially longitudinal follow-up of newly diagnosed, drug-free PCOS patients, will help to separate the independent effects of disease and drugs. In addition, other potential confounding factors, such as the specific dietary structure and lifestyle of the participants, which are not fully understood, may also have an impact on microbiome composition. The role of intestinal microbiota in complex metabolic diseases such as PCOS is a dynamic network system, which is deeply affected by host physiology, genetics, drugs and environment. Further studies with larger samples, longitudinal design, and multi-omics (metagenomics, metabolomics) are needed to fully clarify the direction and mechanism of the causal relationship. Ultimately, such efforts will provide a robust basis for the development of microbiome-based precision diagnostic tools and novel adjunctive therapies (e.g., probiotics, dietary interventions).

## Conclusion

There is a difference in the relative abundance of *Acidimicrobiia* in the gut between PCOS-NIR and PCOS-IR patients before treatment, which is positively correlated with HOMA-IR, suggesting that *Acidimicrobiia* may be involved in the occurrence of PCOS-IR. Drospirenone and ethinylestradiol tablets (II) combined with metformin tablets can change the gut microbiota relative abundance of patients with PCOS-IR, which may be related to the improvement of host lipid and glucose metabolism.

## Data Availability

The original contributions presented in the study are included in the article/**Supplementary Material**. Further inquiries can be directed to the corresponding authors.
