# Supplementary figures and images for: Effects of drospirenone and ethinylestradiol tablets (II) combined with metformin on the composition of gut microbiota in polycystic ovary syndrome with insulin resistance
Source: Front Endocrinol (Lausanne). 2025 Oct 13;16:1581504. doi: 10.3389/fendo.2025.1581504 (PMC12554447; doi:10.3389/fendo.2025.1581504)

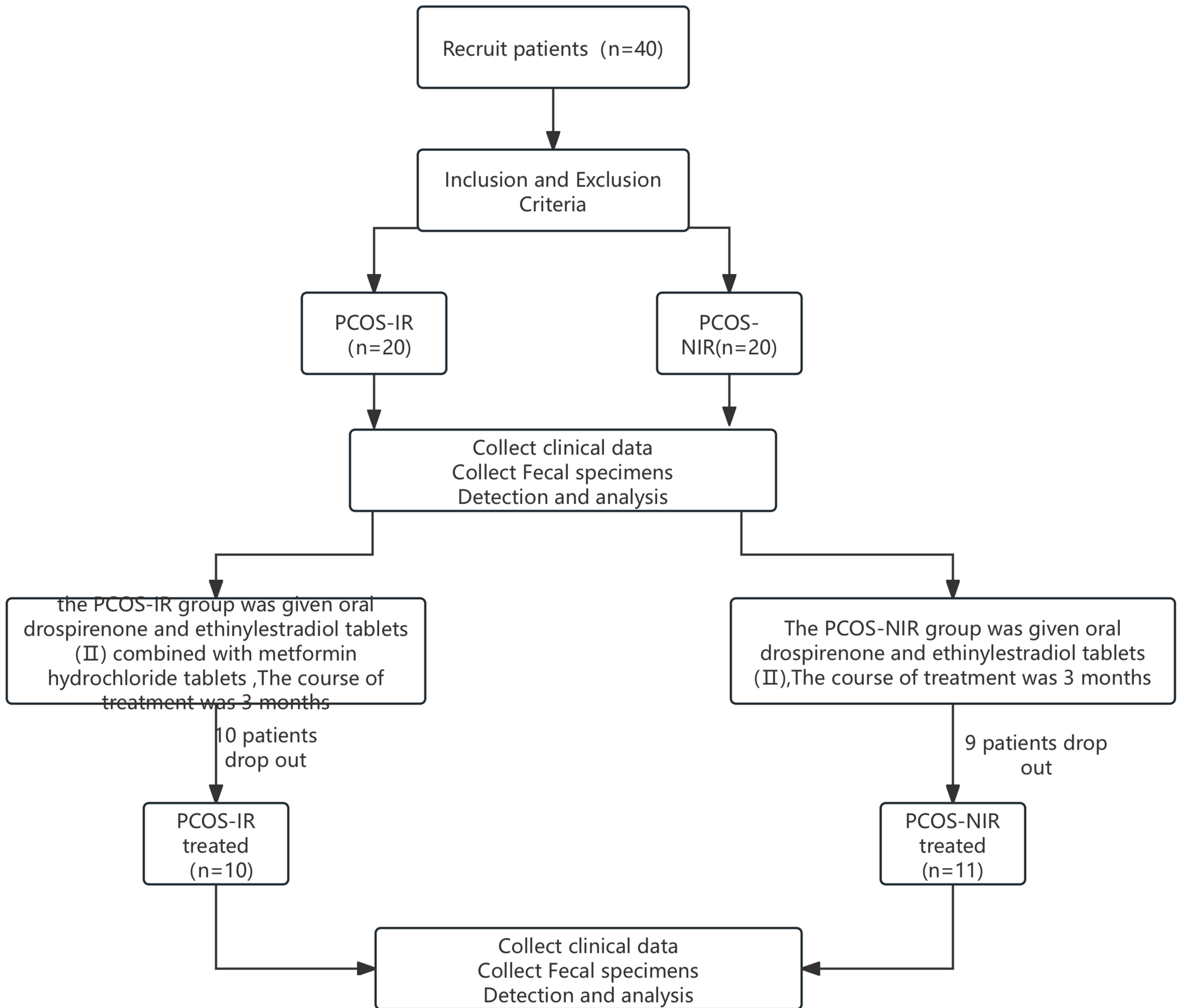

Supplement: Supplementary file 1 [file DataSheet1.pdf]
